# Tissue and Life Stage Specificity of Glutathione S-Transferase Expression in the Hessian Fly, *Mayetiola destructor*: Implications for Resistance to Host Allelochemicals

**DOI:** 10.1673/031.007.2001

**Published:** 2007-04-09

**Authors:** Omprakash Mittapalli, Jonathan J. Neal, Richard H. Shukle

**Affiliations:** ^1^ Department of Entomology, Purdue University, West Lafayette, USA; ^2^ USDA-ARS, Department of Entomology, Purdue University, West Lafayette, USA

**Keywords:** wheat, allelochemicals, mRNA, *Mdes*GST-1, *Mdes*GST-2, *Mdes*GST-3

## Abstract

Two new Delta and Sigma glutathione S-transferases (GSTs) in the Hessian fly, *Mayetiola destructor* (Diptera: Cecidomyiidae), were characterized and transcription profiles described. The deduced amino acid sequences for the two *M. destructor* Delta GSTs (MdesGST-1 and MdesGST-3) showed high similarity with other insect Delta GSTs including the conserved catalytic serine residue. The deduced amino acid sequence for the *M. destructor* Sigma GST (MdesGST-2) showed high similarity with other insect Sigma GSTs including the conserved glutathione and substrate binding sites. Quantitative tissue expression analysis showed that mRNA levels for *MdesGST-1* were predominant in fat body, whereas for *MdesGST-2* and *MdesGST-3* expression was predominant in the midgut. Temporal expression during development showed peak mRNA levels for *MdesGST-1* during larval development, but in the pupal stage for *MdesGST-2. MdesGST-3* showed a constitutive expression pattern throughout development. *M. destructor* feeds on wheat, and expression analysis after feeding indicated that mRNA levels for *MdesGST-1* were significantly higher in incompatible interactions in which larvae fed on resistant wheat, while *MdesGST-3* was significantly higher in compatible interactions when larvae fed on susceptible wheat. *MdesGST-2* showed an equivalent expression pattern during both interactions. These results suggest that the *M. destructor* Delta GSTs are important in detoxifying wheat allelochemicals during feeding, while Sigma GST participates in metabolism of endogenous substrates.

## Introduction

Insects are under constant pressure from toxic substrates generated endogenously or encountered from exogenous sources (Ahmad and Pardini 1990). The major endogenous source of deleterious electrophiles is oxidative metabolism (Fournier et al. 1992). Insect species must overcome the effects of exogenous xenobiotics such as insecticides (Tu and Akgül 2005; Enayati et al. 2005) and prooxidant plant allelochemicals (Ma et al. 1994; Krishnan and Kodrík 2006). In herbivorous insects the midgut hosts complex detoxification mechanisms that include esterases, cytochrome P450S and glutathione S-transferase (GST) enzymes. GSTs neutralize the toxic effects of eletrophilic substrates by conjugating reduced glutathione (GSH) to these compounds producing less toxic, water-soluble substrates (Grant and Matsumura 1989). Thus, these enzymes form an integral part of the phase-II detoxification system (Kostaropoulos et al. 1996). Some GSTs function in hormone biosynthesis and intracellular transport (Enayati et al. 2005).

Insect GSTs were initially classified into two classes based on immunological assays performed on two distinct GSTs first identified and characterized in the house fly *Musca domestica* (Fournier et al. 1992). However, with the advent of more insect genome sequencing, a greater number of similar genes have been deciphered and some of them seem to not fit within these classes (Chelvanayagam et al. 2001; Ranson et al. 2001). Hence, more recently, the classification of insect GST genes has been adopted according to the classification followed in mammalian systems. The members of class I are now categorized as Delta and are insect specific while, members of class II are included in the class Sigma that also include GST genes from other phyla (Enayati et al. 2005).

The Hessian fly, *Mayetiola destructor* (Diptera: Cecidomyiidae) is the major insect pest of wheat worldwide and poses a serious concern in all wheat production areas of the United States. However, the molecular interactions between this pest and its host plant are just now beginning to be revealed (Mittapalli et al. 2005a; 2005b). The first two (feeding) larval instars cause the damage and symptoms of host infestation. These include severe stunting, development of dark green foliage and ultimately death of seedlings (Byers and Gallun 1972). Genetic resistance in wheat cultivars is the best means of control for this destructive insect pest (El Bouhssini et al. 2001).

To date 32 *M. destructor* resistance genes have been identified for possible breeding efforts (Sardesai et al. 2005). Resistance to *M. destructor* attack is via larval antibiosis (Gallun 1977), which is governed by single genes that are completely or partially dominant (Zantoko & Shukle 1997; El Boushini et al. 1998). The deployment of resistant cultivars has led to the appearance of biotypes of the pest that can survive on formerly resistant wheat. Virulent biotypes pose a serious threat for future wheat cultivation (Martin-Sanchez et al. 2003). Thus, there is a need to identify alternative targets within the *M. destructor* that could affect its survival on wheat seedlings.

There are two distinct interactions of the *M. destructor* with wheat based on the survival of *M. destructor* larvae. A compatible interaction involves first instar larvae that can successfully feed and develop on a susceptible wheat seedling. During an incompatible interaction first instar larvae on resistant wheat plants are prevented from establishing a sustained feeding site and die within a period of five days after hatching (Grover et al. 1988). Wheat plants in incompatible interactions undergo little or no physiological stress.

Yoshiyama and Shukle (2004) reported the identification and characterization of a Delta GST (*MdesGST-1*) in the *M. destructor*. We report here the characterization of two additional *M. destructor* GSTs, *MdesGST-2* and *MdesGST-3*, that fit into the Delta and Sigma classes, respectively. Both GSTs were recovered from a *M. destructor* midgut expressed sequence tag database. Only these three GSTs have been identified in the *M. destructor* although the genome may encode other classes. mRNA for all three *M. destructor* GSTs was quantified in tissues, during development, and in larvae fed on susceptible and resistant wheat plants. Results obtained in this study revealed that the *M. destructor*, as in other Diptera (*Drosophila melanogaster* and *Anopheles gambiae*), has at least two different classes of GSTs, which may function in defense against allelochemicals during feeding of first and second instars, and/or reactive oxygen species encountered in the third instar, pupae and adults.

## Materials and Methods

### Insect and plant material

*M. destructor* Biotype L was used in this study and was maintained as described by Sosa and Gallun (1973). To date sixteen *M. destructor* biotypes have been identified and are designated GP and A to O (Ratcliffe et al. 1994). Biotype L was established from a field collection made from Posey County, Indiana in 1986. All biotypes were selected according to the methods described by Sosa and Gallun (1973). Biotype L was reared on ‘Newton’ wheat, which carried no genes for resistance, and on ‘Iris’ wheat, which carries the resistance gene *H9*. These wheat lines were seeded in 10-cm pots (∼20 seeds/pot) filled with soil and grown in flats contained in chambers at 20°C with a 12-h photoperiod. Infestation was performed by covering the flats with a tent of nylon mesh and allowing females to oviposit on the seedling plants at the one-leaf stage. Egg hatch normally takes 3 to 4 days after oviposition depending on temperature. First, second and third instar larvae and pupae were collected by dissecting the crown portions of infested wheat seedlings in water and immediately flash-frozen in liquid nitrogen. Adults were collected after emergence, cold anesthetized, and flash-frozen in liquid nitrogen. All the samples were stored at -80°C until RNA was isolated.

### Larval dissections and RNA extraction

Three hundred late first instar larvae (five days after egg hatch) were dissected in ice-cold Schneider's insect medium (Sigma-Aldrich, www.sigmaaldrich.com). Larval midgut, salivary glands and fat body tissues were isolated as described previously (Grover et al. 1988; Mittapalli et al. 2005b) and placed in 100 µ of ice-cold Schneider's in separate 1.5 ml micro-centrifuge tubes. Immediately following collection, the tissues were flash-frozen in liquid nitrogen and stored at -80°C until RNA was isolated. Total RNA was isolated from the dissected tissues using the RNAqueous®-4PCR kit from Ambion (Austin, TX) following the manufacturer's protocol.

### Construction of midgut cDNA libraries

RNA extracted from the midgut tissue was used to construct a cDNA library using a SMART ™ cDNA library construction kit from BD Biosciences (www.bdbiosciences.com) following the manufacturer's protocol with one modification (Mittapalli et al. 2005b); the cDNAs obtained were not cloned into the phage vector supplied with the kit but rather directly into the PCR®4-TOPO® vector included in a TOPO TA cloning® for sequencing kit (Invitrogen, www.invitrogen.com). Plasmid DNA was isolated using a Qiagen BioRobot 3000 (www.qiagen.com) and cloned DNA expressed sequence tag fragments were sequenced from the 5′ end by the Purdue Genomics Center using a primer designed to the 5′ cloning oligonucleotide of the cDNA library construction kit.

### Sequence comparison and alignment

Annotations and sequence similarity for the recovered GSTs were done using the basic local alignment search tool (BLAST) programs on the National Center for Biotechnology Information (NCBI, Bethesda, MD, http://www.ncbi.nlm.nih.gov/). Sequence alignments were performed using the GENETYX-MAC 10.1 software (Software Development Co., Ltd., Tokyo, Japan). Conserved domains (CD-search) in the deduced amino acid sequences of the *M. destructor* GSTs were revealed using the BLAST search engine on the NCBI browser (Marchler-Bauer and Bryant 2004).

### Analyses of the *M. destructor* GSTs in larval tissues and during development

The larval tissues collected from first and early second instars, as described above, were pooled into three categories, midgut, salivary glands and fat body and the RNA extracted from each pool was used to determine the transcript levels of the GSTs in the tissues by quantitative real-time PCR (see below). Total RNA was also isolated from all the life stages of the *M. destructor* including first instars (four days after hatch), second instars (eight days after hatch), third instars, pupae and adults to determine the transcript levels of the GSTs during development.

### Analyses of the *M. destructor* GSTs during compatible and incompatible interactions

A compatible interaction was represented by Biotype L larvae on susceptible Newton wheat seedlings. An incompatible interaction was represented by Biotype L larvae on resistant Iris seedlings. Total RNA was extracted from 1–4 day-old larvae from both interactions to determine the transcript levels of the GST genes in larvae during compatible and incompatible interactions.

### Quantitative analysis

Quantitative real-time PCR was used to assess transcript levels of *MdesGST-1*, *MdesGST-2* and *MdesGST-3* in tissues, during development and in larvae on susceptible and resistant plants. The software Primer Express from Applied Biosystems (http://www.appliedbiosystems.com/) was used to design the real-time primers. The primer sequences for these analyses are shown in Table 1. In brief, the single-strand cDNA synthesis was performed as follows: 1 µg of total RNA in 10 µl of water was treated with DNase using the DNA-free kit (Ambion www.ambion.com) following the manufacturer's instructions. The reverse transcriptase reaction to generate the cDNA for use in quantitative real-time PCR was carried out using the Superscript First Strand cDNA Synthesis kit (Invitrogen) as follows: 1 µl of oligo d(T) primer and 1 µl of dNTPs were added to the 10 µl of total RNA. The mixture was heated at 65°C for 5 min and then placed on ice. The following were added on ice: 2 µl of 10X first strand buffer, 2 µl of 50 mM MgCl_2_, 2 µl of 0.1 M DTT, 1 µl of RNaseOut, and 1 µl of Superscript II reverse transcriptase. cDNA synthesis was performed by at 42°C for 2 h. Reactions were stopped by heating samples at 70°C for 15 min.

**Table 1.**
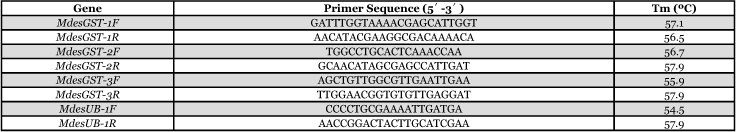
Primer sequences used in the quantitative real-time PCR

Quantification of cDNA, displayed as relative expression value (REV) was based on the Relative Standard Curve method (User Bulletin #2: ABI Prism 7700 Sequence Detection System http://docs.appliedbiosystems.com/pebiodocs/04303859.pdf) using serial dilutions of a cDNA sample containing the target sequence. After quantitative real-time PCR amplification the threshold cycle (Ct) value for each dilution was plotted against the log of its concentration, and Ct values for the experimental samples were plotted onto this dilution series standard curve. Target quantities were calculated from separate standard curves generated for each experiment. REVs were then determined by dividing the quantities of the target sequence of interest with the quantity obtained for ubiquitin. The entire analysis was performed using a *M. destructor* ubiquitin (DQ674274) as an internal standard (reference), which in our evaluations has shown constant expression in the *M. destructor* during development. Ubiquitin has been shown to be a suitable internal reference in a number of experimental systems (Jin et al. 2003; Luo et al. 2005; Yuan et al. 2006). PCR cycling parameters included 50°C for 2 min, 95°C for 10 min, and 40 cycles of 95°C for 15 sec, and 60°C for 1 min.

### Statistical analysis

For calculations of significance, the logs of the REVs for each gene were analyzed by Analysis of Variance using the PROC MIXED procedure of SAS (SAS Institute Inc. SAS/STAT User's Guide, Version 9.1). For the expression analysis pertaining to tissues and developmental stages, the statistical model included treatment and interaction between treatments whereas for the analysis of expression in different larval *M. destructor*/wheat interactions (compatible versus incompatible), the statistical model included treatment, time points, and interaction between treatments and time points as fixed effects. Biological replicates were included as a random effect in the analysis model. Treatment differences at each time point were evaluated using orthogonal contrasts and considered statistically significant if the p-value associated with the contrast was p< 0.05.

Relative fold change in the case of tissue expression was calculated by taking the salivary gland mRNA experimental samples as the calibrator sample (confer User Bulletin #2: ABI Prism 7700 Sequence Detection Syste, see below). Hence, the fold changes in the *M. destructor* GST transcripts in the midgut and fat bodies were calculated relative to the salivary glands. During development, expression in the first instars was taken as the calibrator. Fold change in gene expression for *MdesGST-1* during compatible and incompatible interactions was assessed by dividing the REV for larvae on susceptible plants by the REV for larvae on resistant plants. For *MdesGST-2* and *MdesGST-3* fold changes during these interactions was determined by dividing the REV for larvae on resistant plants by the REV for larvae on susceptible plants for each of the four time points examined. For the tissue/developmental analysis three technical replicates were used, whereas, for the analysis during different interactions three biological replicates (two technical replicates within each) were used. The standard error represented the variance in these biological replicates for the respective analysis.

**Figure 1.**
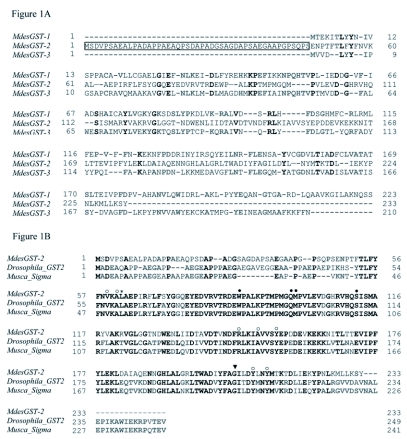
Alignments of the deduced amino acid sequences. (A) Homology shared among all three *Mayetiola destructor* GSTs, MdesGST-1 (AAS00666), MdesGST-2 (DQ662542) and MdesGST-3 (DQ662543). The boxed sequence of MdesGST-2 represents the N-terminal extension constituted by a hydrophobic/acidic motif. (B) Homology of MdesGST-2 with sequences from other insect Sigma GSTs: *Musca domestica* (P46437), and *Drosophila melanogaster* (AAM48357). Filled and open circles represent residues that constitute the putative glutathione (GSH) and electrophilic-substrate binding sites, respectively. The putative H-site residue of MdesGST-2 (L62), which also contacts GSH, is indicated by an asterisk. The bulge-inducing residue (G205) of MdesGST-2 is marked with an inverted filled triangle, [*continued on next page*]

## Results

### Characterization of the *M. destructor* GSTs

Results from blastp homology searches indicated that the *M. destructor* genome encodes both Delta (*MdesGST-1* and *MdesGST-3*) and Sigma (*MdesGST-2*) classes of GSTs. The nucleotide and deduced amino acid sequences for MdesGST-1 were reported by Yoshiyama and Shukle (2004). Therefore, this study was confined to the characterization of *MdesGST-2* and *MdesGST-3*. The deduced amino acid sequence for MdesGST-2 was characterized with an n-terminal extension commonly found in other Sigma GSTs (Figure 1A). The deduced amino acid sequence for MdesGST-2 revealed 76% identity at a 5e-77 threshold with a Sigma GST from the housefly (*Musca domestica*, P46437) and 72% identity at a 6e-77 threshold with a GST2 from the fruit fly (*D. melanogaster*, AAM48357) (Figure 1B). Homology searches for MdesGST-3 at the amino acid level revealed 71% identity (2e-88 threshold) and 69% identity (2e-86 threshold) with Delta GSTs from the African malaria mosquito (*A. gambiae*, 40889324) and the marmalade hoverfly (*Episyrphus balteatus*, CAH58743), respectively (Figure 1C). The nucleotide sequences for *MdesGST-2* and *MdesGST-3* were submitted to GenBank with accession numbers DQ662542 and DQ662543, respectively.

**Figure 1.**
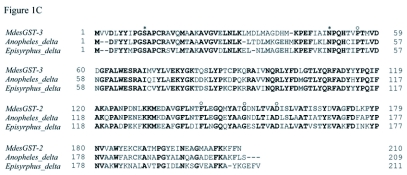
[*continued*] (C) Homology of MdesGST-3 with sequences from other insect Delta GSTs: *Anopheles gambiae* (40889324), and *Episyrphus balteatus* (CAH58743). S11 and N49 of MdesGST-3, which represent the catalytic pocket, are indicated with an asterisk. Amino acid residues determining folding are marked with open circles. In all three panels, identical residues among all taxa are highlighted in black and identical residues in two of the three taxa are highlighted in gray. Gaps in the alignment are indicated by dashes.

The alignments of deduced amino acid sequences between MdesGST-2 and other insect Sigma GSTs indicated several conserved residues across the insect species under comparison. These residues constituted the putative GSH binding site (Y56-W87-Q98-S112-) and the electrophilic-binding site (V59-A61-R148-A152-Y156-Y211-Y214) in the deduced amino acid sequence for MdesGST-2 (Figure 1B). L62 represented the putative H-site residue, which contacts GSH. G205 indicated the bulge-inducing residue (Agianian et al. 2003). Similarly, the alignment of deduced amino acid sequences between MdesGST-3 and other insect Delta GSTs also revealed key residues conserved across all the insect species (Figure 1C). The deduced amino acid sequence for MdesGST-3 contained S11, the catalytic residue used by Delta GSTs and P55-L143-G151-D158, which determine folding of the GSTs molecules.

### Tissue-specific expression patterns of the *M. destructor* GSTs

**Figure 2.**
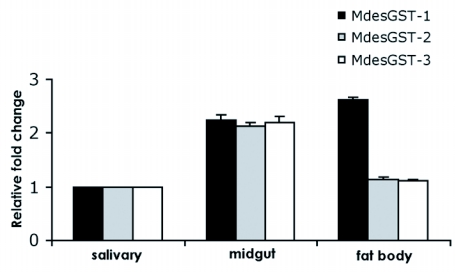
Transcriptional patterns of the *M. destructor* GSTs in larval tissues. Data shown represents relative fold changes in the expression of *MdesGST-1*, *MdesGST-2*, and *MdesGST-3* in midgut, salivary glands and fat body tissues. Expression in the salivary glands was taken as the calibrator and the expression in midgut and fat body samples was calculated relative to the expression in the salivary glands. The standard error is represented by the error bar for three technical replicates. Asterisks indicate fold changes in expression significantly different (p>0.05) relative to salivary glands.

Quantitative analysis of the *M. destructor* GST transcripts in larval tissues including midgut, salivary glands and fat body suggested their mRNA abundance to be tissue-specific in expression. The greatest levels of mRNA for *MdesGST-1* were observed in the fat body and midgut, whereas the mRNA levels for *MdesGST-2* and *MdesGST-3* were predominant in the midgut (Figure 2). The least level of transcriptional expression for all the *M. destructor* GSTs was found in the salivary gland samples and thus the expression in midgut and fat body were compared relative to the salivary glands. A significant (p< 0.05) fold difference of 2.6 and 2.3, respectively, was calculated for *MdesGST-1* in the fat body and midgut samples relative to the salivary gland tissue (Figure 2). Further, a fold change of 2.1 for *MdesGST-2* and 2.2 for *MdesGST-3* was calculated between the midgut and salivary gland tissues.

### Developmental expression patterns of the *M. destructor* GSTs

**Figure 3.**
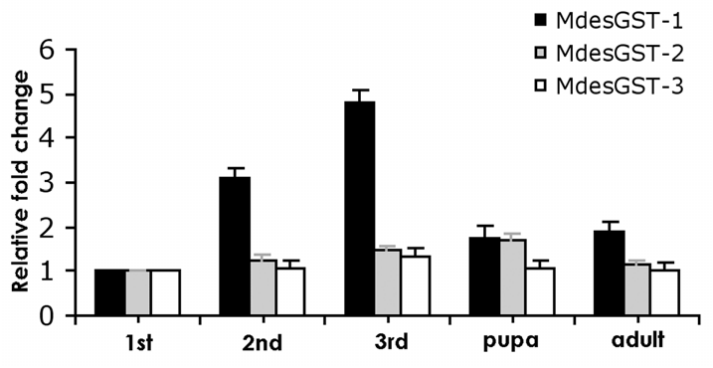
Transcriptional patterns of the *M. destructor* GSTs during development. Data presented reveals the fold-changes. For all the genes analyzed, the first instar was taken as the calibrator sample. The standard error is represented by the error bar for three technical replicates. Asterisks indicate fold changes in expression significantly different (p>0.05) relative to the first instar.

Transcription profiling for the *M. destructor* GST genes was also performed for all the stages of development including the three larval instars, pupae and adults. Of all three *M. destructor* GSTs, mRNA for *MdesGST-1* was observed to be the most abundant, while mRNA for *MdesGST-3* was the least abundant. *MdesGST-1* showed an ascending pattern in mRNA levels during the larval instars (Figure 3), with a peak in the third instars. Interestingly, the expression profile for the Sigma GST (*MdesGST-2*) revealed a peak mRNA level in pupae (Figure 3). The lowest level of expression for all three *M. destructor* GSTs was observed in the first instar samples. Therefore, the fold change in mRNA abundance in the other developmental stages was calculated relative to this basal level in the first instars. Significant (p<0.05) fold differences of 3.2, 4.8, 1.8 and 1.9 for *MdesGST-1* were determined between second instar, third instar, pupa, and adult respectively compared to the first instar, while a 1.7-fold difference (p<0.05) for *MdesGST-2* was calculated between pupa and first instar (Figure 3). Levels of mRNA for *MdesGST-3* did not significantly vary throughout development.

### Expression patterns of the *M. destructor* GSTs during interactions with wheat

**Figure 4.**
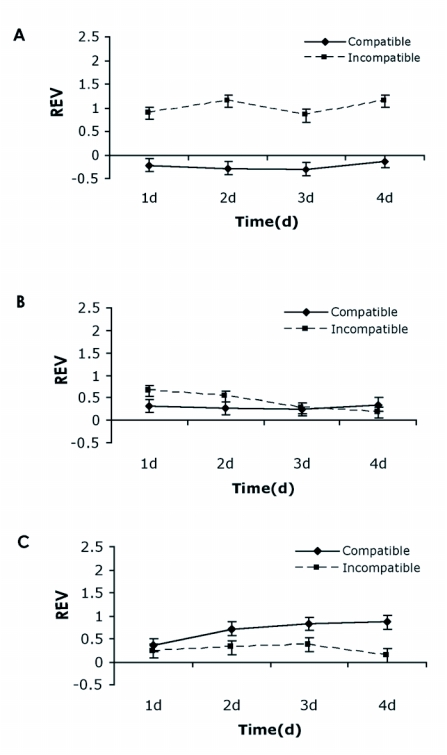
Differential expression patterns of the *M. destructor* GSTs during compatible and incompatible interactions with wheat. Quantitative analysis was performed using RNA samples from larvae participating in compatible interactions (Biotype L Hessian flies on susceptible ‘Newton’ wheat) and from larvae participating in incompatible interactions (Biotype L Hessian flies on resistant ‘Iris’ wheat) 1 through 4 days (d) post hatch. Values represent the Log of the mean relative expression value (REV) plotted against each of the four time points examined for (A) *MdesGST-1* (B) *MdesGST-2* and (C) *MdesGST-3*. REVs for all genes were calculated relative to the expression of a *M. destructor* ubiquitin gene. The standard error in all panels is represented by the error bar for two biological replicates (two technical replicates within each).

It was also of interest to determine transcriptional expression patterns for the *M. destructor* GST genes in larvae on susceptible and resistant wheat plants during compatible and incompatible interactions. All three *M. destructor* GSTs showed different expression patterns during these interactions. The mRNA level for *MdesGST-1* was significantly (p < 0.05) up-regulated in larvae on resistant wheat plants compared to similar aged larvae on susceptible wheat plants for 1–4 day-old larvae (Figure 4A). The greatest fold change of 8.1 for MdesGST-1 was observed between 4 day-old larvae (data not shown). Quantitative analysis for *MdesGST-2* suggested modest levels of changes in the mRNA levels of larvae infesting resistant wheat plants. Only the first two time points (land 2 day-old larvae), were significantly different (p< 0.05), but the later time points were not (Figure 4B). This observation was further supported by the fold-change data, which indicated a fold-difference of less than 1.0 for 3 and 4 day-old larvae (data not shown). In contrast to the transcriptional expression patterns observed for *MdesGST-1* and *MdesGST-2*, the mRNA level for *MdesGST-3* was found to be significantly (p< 0.05) up-regulated in 2, 3 and 4 day-old larvae on susceptible wheat compared to similar aged larvae on resistant wheat (Figure 4C). The greatest difference in the mRNA level for *MdesGST-3* was observed between 4 day-old larvae, which corresponded to a 7.2 fold-change (data not shown).

## Discussion

### *M. destructor* Delta and Sigma GSTs

Two new glutathione S-transferases, *MdesGST-3* and *MdesGST-2*, were characterized and transcription profiles described. In addition, the transcriptional expression patterns of another GST gene, *MdesGST-1*, previously reported by Yoshiyama and Shukle 2004 is described. Of the three *M. destructor* GST genes, two of them fall within the Delta class (*MdesGST-1* and *MdesGST-3*) and the third within the Sigma class (*MdesGST-2*). This classification of the *M. destructor* GSTs was primarily based on the identity shared at the amino acid level with other known insect GSTs belonging to similar classes. The deduced amino acid sequences for the *M. destructor* GSTs were in agreement in length and contained conserved GST domains that are characteristic of other insect Delta GST amino acid sequences (Chen et al. 2003) and Sigma GSTs (Agianian et al. 2003).

Insect genomes contain diverse GST genes that share functionality with those present in lower and higher animals (Enayati et al. 2005). The complexity of GST genes found within an insect species is best exemplified by the genomes of *D. melanogaster* (Toung et al. 1993; Tu and Akgul 2005), *A. gambiae* (Ranson et al. 2001; Ranson and Hemingway 2005) and *M. domestica* (Fournier et al. 1992; Zhou and Syvanen 1997), which are characterized by multigene GST families. In the genomes of two dipteran species, *D. melanogaster* and *A. gambiae*, only a single putative transcript of the Sigma GST has been identified, while more than 10 putative transcripts encoding for Delta GSTs have been identified (Enayati et al. 2005). However, it has been documented in *A. gambiae* that both the Sigma and Delta classes of GSTs exist as alternative splice variants (Ding et al. 2003; Ranson et al. 1998).

### Induction of MdesGST-1 and MdesGST-3 mRNAs

The mRNA transcripts for MdesGST-1 and MdesGST-3 were found in all the tissues examined (midgut, fat body and salivary glands). This observation is in agreement with the results obtained by Yoshiyama and Shukle (2004) for *MdesGST-1* with reverse transcription PCR. However, quantitative analysis performed in this study revealed the greatest mRNA levels for *MdesGST-1* in the fat body and midgut and for *MdesGST-3* in the midgut. Spatial expression studies in *D. melanogaster* showed that the gut epithelium consisted of three expression domains which were essential for the expression of *Gst-D1* (Nakamura et al. 1999). Several other studies have also reported that the midgut plays an important role in GST-based detoxification in an array of herbivorous insect species including members of Lepidoptera (Snyder at l. 1995; Periæ-Mataruga et al. 1997; Krishnan and Kodrík 2006), Diptera (Nakamura et al. 1999) and grasshoppers (Konno and Shishido 1992; Barbehenn 2002). GST expression has also been observed in other tissues of insects such as Malphigian tubules (Konno and Shishido 1992), fat body (Konno and Shishido 1992; Snyder et al 1995) and antennae (Rogers et al. 1999). The peak expression obtained for *MdesGST-1* in fat body corroborates with the findings of Konno and Shishido (1992) and Snyder et al., (1995). The higher mRNA levels for *MdesGST-1* and *MdesGST-3* in the midgut of the *M. destructor* larvae suggests a detoxification mechanism that could be associated with the allelochemicals present in their diet. The low mRNA levels observed in other tissues suggest additional physiological functions of the GST proteins in the biology of the *M. destructor*.

An intriguing expression pattern was observed in larvae during compatible and incompatible interactions with wheat. In a compatible interaction, larvae on resistant wheat plants larvae were able to continuously feed and could be challenged with wheat allelochemicals during the feeding event (Mittapalli et al. 2005b). However, in an incompatible interaction, larvae failed to establish a sustained feeding site and thus underwent severe stress resulting in death within 5 days (Painter 1930). In the current study it was found that the mRNA level for *MdesGST-1* was significantly greater in larvae in incompatible interactions. The greater abundance of *MdesGST-1* mRNA in larvae on resistant wheat could be explained by the response of induced plant defense compounds such as flavonoids (Hodnick et al. 1989) in resistant wheat plants. Further, endogenous sources of reactive oxygen species stemming from stress within the larvae could contribute to the induction of *MdesGST-1*. The high level of *MdesGST-1* mRNA in the fat body supports the latter explanation.

Insect Delta GSTs have a key role in detoxifying xenobiotics including insecticides and plant allelochemicals. In this study, *MdesGST-3* was induced at modest levels in larvae in compatible interactions. *MdesGST-3* is a target for future studies of detoxifying wheat allelochemicals during feeding on susceptible wheat plants.

The level of induction of GSTs in other insects in response to feeding is mixed. Several studies report a significant induction of GST activity in larvae fed on natural plant diet containing prooxidant allelochemicals (Egaas et al. 1992; Vanhaelen et al. 2003; Krishnan and Kodrík 2006) or fed an artificial diet supplemented with plant allelochemicals (Wadleigh and Yu 1988; Lee 1991). However, Yu (1989) found a moderate GST activity in fall armyworms (*Spodoptera frugiperda*) upon ingestion of xanthotoxin and Snyder et al., (1995) reported a moderate level of mRNA induction for a Sigma GST when *Manduca sexta* was fed dietary chemicals.

### Putative functions of MdesGST-2

The functions of Sigma GSTs in insects remain unclear (Agianian et al. 2003). Sigma GSTs in *D. melanogaster* had been initially reported to have structural functions in the indirect flight muscle tissues (Bullard et al. 1988; Clayton et al. 1998). More recently, the Sigma GST homolog in *D. melanogaster* (DmGSTS-1) was assigned (alternative) putative functions related to detoxification, anti-oxidant defense and cell signaling processes (Singh et al. 2001; Agianian et al. 2003).

mRNA levels for *MdesGST-2* were highest in the midgut. This is in agreement with the mRNA levels associated with a Sigma GST recovered from a larval midgut cDNA library of *M. sexta* (Snyder et al. 1995). Additionally, a low level of *M. domestica* GST-2 was observed in non-muscular tissues (Franciosa and Berge 1995). The *M. destructor* Sigma GST, *MdesGST-2*, could participate in detoxification/anti-oxidant functions similar to those observed in *D. melanogaster* based on the developmental expression pattern. Unlike the two *M. destructor* Delta GST transcripts (*MdesGST-1* and *MdesGST-3*), the peak mRNA level for *MdesGST-2* was observed in pupae. This result is similar to the findings in the yellow mealworm, *Tenebrio molitor* (Kostaropoulos et al. 1996). Up-regulation of *MdeGST-2* in pupae could be required as a response to increased metabolism related to morphological changes during this stage (Kostaropoulos et al. 1996). As a result of these alterations, the action of anti-oxidant enzymes is essential to counteract the toxic effects of electrophilic substrates generated during this massive re-organization process. Indeed, this seems to be the most plausible explanation also given for another dipteran, *A. gambiae* (Hazelton and Lang 1983). Further, the mRNA levels for *MdesGST-2* during compatible and incompatible interactions remain equivalent. There is no basis for suggesting it is involved in detoxifying wheat allelochemicals.

## Conclusions

The *M. destructor* genome codes for at least two classes of GST genes. The product of the Delta GST genes may aid in detoxifying exogenous allelochemicals from its host plant, while that of a Sigma GST could function in protection against toxic oxygen species generated endogenously during development. These results provide a better understating of the mechanisms employed by the *M. destructor* during its interactions with wheat. Proteomic studies are needed to determine the functions of these enzymes.

## Abbreviations

MdesGST-1,: *Mayetiola destructor* glutathione S-transferase-1
MdesGST-2,: *Mayetiola destructor* glutathione S-transferase-2
MdesGST-3,: *Mayetiola destructor* glutathione S-transferase-3
REV,: relative expression value
